# Synergistic interplay between melatonin and hydrogen sulfide enhances cadmium-induced oxidative stress resistance in stock (*Matthiola incana* L.)

**DOI:** 10.1080/15592324.2024.2331357

**Published:** 2024-04-02

**Authors:** Faisal Zulfiqar, Anam Moosa, Hayssam M. Ali, John T. Hancock, Jean Wan Hong Yong

**Affiliations:** a Department of Horticultural Sciences, Faculty of Agriculture and Environment, The Islamia University of Bahawalpur, Bahawalpur, Pakistan; b Department of Plant Pathology, Faculty of Agriculture and Environment, The Islamia University of Bahawalpur, Bahawalpur, Pakistan; c Department of Botany and Microbiology, College of Science, King Saud University, Riyadh, Saudi Arabia; d School of Applied Sciences, University of the West of England, Bristol, UK; e Department of Biosystems and Technology, Swedish University of Agricultural Sciences, Alnarp, Sweden

**Keywords:** Antioxidants, oxidative stress markers, sugars, MLT, H_2_S, cadmium tolerance

## Abstract

Ornamental crops particularly cut flowers are considered sensitive to heavy metals (HMs) induced oxidative stress condition. Melatonin (MLT) is a versatile phytohormone with the ability to mitigate abiotic stresses induced oxidative stress in plants. Similarly, signaling molecules such as hydrogen sulfide (H_2_S) have emerged as potential options for resolving HMs related problems in plants. The mechanisms underlying the combined application of MLT and H_2_S are not yet explored. Therefore, we evaluated the ability of individual and combined applications of MLT (100 μM) and H_2_S in the form of sodium hydrosulfide (NaHS), a donor of H_2_S, (1.5 mM) to alleviate cadmium (Cd) stress (50 mg L^−1^) in stock (*Matthiola incana* L.) plants by measuring various morpho-physiological and biochemical characteristics. The results depicted that Cd-stress inhibited growth, photosynthesis and induced Cd-associated oxidative stress as depicted by excessive ROS accumulation. Combined application of MLT and H_2_S efficiently recovered all these attributes. Furthermore, Cd stress-induced oxidative stress markers including electrolyte leakage, malondialdehyde, and hydrogen peroxide are partially reversed in Cd-stressed plants by MLT and H_2_S application. This might be attributed to MLT or H_2_S induced antioxidant plant defense activities, which effectively reduce the severity of oxidative stress indicators. Overall, MLT and H_2_S supplementation, favorably regulated Cd tolerance in stock; yet, the combined use had a greater effect on Cd tolerance than the independent application.

## Introduction

Ornamental crops are susceptible to heavy metals (HMs), hence global expansion of these metals may hamper the horticulture and ornamental sectors.^1,2^ Cadmium, one of the potential harmful metals that enters into the soils from industrial and agricultural sources, is considered hazardous, harmful, and toxic.^3,4^ It is water soluble and mobile in nature.^4^ Plants do not require Cd and excessive amounts of Cd in plants may impair their metabolic activities.^5^ Plants have developed different adaptive mechanisms including redox homeostasis, extracellular barriers in the cell wall, biosynthesis of chelates, and the transmission of secondary signals (such as hormonal, metabolic, hydrogen sulfide, and nitric oxide to deal with Cd stress).^4,5^ Plants exposed to Cd have a number of abnormalities, including stunted development, chlorosis of the leaf, and disruption of several critical biological processes including photosynthesis, water balance, mineral absorption, defense enzymes, membrane stability, gene expression and DNA structure.^6^ Cadmium in the growing media enters through the roots and excessive reactive oxygen species (ROS) burst happens when it enters at an excessive rate. Numerous key cellular elements like lipids, proteins, and DNA are severely harmed by the excessive ROS that are the main drivers of the oxidative stress induced by Cd stress.^4^ The deleterious consequences of Cd stress are further exacerbated by interfering with the intake of some crucial nutrients, such as zinc and copper.^7^

Plants in harsh environments incorporate higher amounts of non-enzymatic antioxidants and enzymatic antioxidants, as well as enzymes associated with the AsA-GSH cycle in order to minimize the harmful effects of ROS.^8^ These protective mechanisms work in harmony to shield the plant from oxidative stress that can harm cells and interfere with normal physiological functions.^9^ Therefore, the regulation of antioxidant activity determines how long plants can survive and at what HMs concentration.^10^ Stock cut flower crop was chosen for the current investigation because it does not consistently use this strategy. Most often, the activated defense mechanism causes growth to be stopped, which ultimately reduces its flower quality and output. To address this, work has been done to improve plant growth, reduce the effects of Cd stress, and understand how Cd stress affects ornamental plants.

Chemically, melatonin (MLT) is known as N-acetyl-5-methoxytryptamine and is found in both plants and mammals. It is an ecologically stable biomolecule. Using beneficial biomolecules as crop protectant agents might allow the plants to better tolerate abiotic stress conditions.^11–13^ Plant MLT is thought to be involved in regulating germination, photosynthesis, flowering, leaf senescence, root development, carbohydrate metabolism, and circadian rhythm as an antioxidant and growth regulator.^14^ Recent research has demonstrated that MLT application on HMs stressed plants may eliminate excess ROS and thus provide relief from the HMs induced oxidative stress in plants that causes growth and yield restrictions (Chen et al. 2023).^11^

To counteract Cd stress, a relatively new research domain focused on innovative signaling molecules has attracted a lot of attention.^17^ The use of signaling molecules as soil, plant or seed application has been investigated as a potential remedy for the detrimental effects of HMs toxicity on plants.^18^ Application of H_2_S is reported to improve the heavy metal tolerance in crop plants such as tomato^13^ and tomato^19^ etc. Notably, numerous studies have demonstrated that MLT can regulate plant growth and HMs stress responses through interactions with other signaling molecules such as H_2_S.^11,20^ The interactive effects of MT and H_2_S were evaluated by Haghi et al.^20^ and found that MLT and H_2_S interaction provided relief from lead (Pb) induced oxidative stress in safflower plants by reducing the Pb uptake and regulating the ascorbate-glutathione cycle.^18^ evaluated that H_2_S induces arsenic tolerance in pepper plants via regulating endogenous MLT level. However, the interactive effects of MLT and H_2_S on Cd tolerance in stock flower have not yet been investigated.

There are not any reports on the combined effect of MLT and H_2_S on Cd stressed stock. It was hypothesized that both these protectants could better ameliorate Cd induced toxicity in ornamental stock. The objectives associated with this experiment were to investigate how these protectants affected physio-biochemical aspects. This study provides a fresh perspective on the mechanisms by which MLT and H_2_S combined application can reduce Cd stress in stock.

## Material and methods

### In planta assay

The agricultural land top layer soil “0–25 cm depth” at the research area of the department of horticultural sciences, faculty of agriculture and environment, The Islamia University of Bahawalpur, Pakistan, was collected for the experiment. Collected soil was sieved (2 mm pore size), and kept in open air for drying before being analyzed. Soil pH and electrical conductivity (EC) were evaluated using McLean’s technique,^21^ and Page et al.,^22^ respectively. According to Bouyoucos’ method,^23^ the hydrometer was used to assess the soil textural analysis. Soil organic content was determined via an established method by Walkley and Black,^24^ as well as total Cd concentration by following Soltanpour.^25^ The results of the soil initial characterization are presented in Table 1.

**Table 1.** Physicochemical characteristics of soil used as a growing media in pot experiment.

Parameters	Value
Texture	Sandy loam soil
Sand	49%
Silt	33%
Clay	18%
pH	7.2
Organic matter	0.65%
Electric conductivity (dS m^−1^)	2.11
Total Cd (mg Kg^−1^)	0.15

### Experimental setup

A pot study was started under natural environmental conditions. Healthy and uniform seeds of stock (*Matthiola incana* L cv. PanAmerican) were obtained from a local seed distributor (Sunny Seeds Company) in Lahore, Pakistan. Seeds were sterilized for 15 min using 4% (w/v) sodium hypochlorite. Seeds were then washed with distilled water four times and germinated in seedling trays filled with peat and perlite (70:30 v/v) based growing media. Seedlings (7 d old) were transferred into the pots filled with 3 kg of soil. The soil was drenched with 50 mg L^−1^ of Cd 15 d after transplanting. Cadmium nitrate was used as Cd source. This concentration was chosen following the morpho-physiological results of our initial experiment (Data not shown). After 4 d of Cd treatment, H_2_S and MLT (Sigma-Aldrich, St. Louis, MO, USA) that were chosen based on the results of the preliminary experiment, were applied to the plants. Treatment details: CK (Control); Cd (50 mg L^−1^), Cd+ H_2_S (50 mg L^−1^ +1.5 mM sodium hydrosulfide (NaHS), a donor of H_2_S), Cd+MLT (Cd 50 mg L^−1^ +100 μM melatonin) and Cd+H_2_S+MLT (Cd 50 mg L^−1^ +1.5 mM NaHS +100 μM melatonin). NaHS application by root irrigation and simultaneous MT foliar spraying was applied at four d intervals, while the nutrient solution pH was adjusted to 6.5. Hoagland solution (0.5X) was used to irrigate the ornamental stock seedlings.

### Vegetative features

Plants were carefully uprooted at termination, washed with distilled water to remove soil, and separated into shoots and roots. Materials were dried in an electric oven for 2 days, and dry weights of roots and shoots were recorded using an electric balance.

### Photosynthetic pigments and leaf gas exchange

Fresh leaf material was promptly chopped into 0.5 cm^2^ pieces using scissors, and samples (≈0.5 g per plant) were extracted with 10 mL 80% (v/v) acetone after holding them at 4°C for 12 h. Centrifugation of the extract was done at 10,000 *g* for 10 min at room temperature. Spectrophotometric absorbance was measured at 645, and 663 nm to evaluate the Chl *a*, and Chl *b*, respectively. Chlorophyll was evaluated following the formula devised by:^26^

- Chl_a_ = (13.95 × A_665_ −6.88 × A_649_) × 0.01/leaf fresh weight
- Chl_b_ = (24.96 × A_649_ −7.32 × A_665_) × 0.01/leaf fresh weight

Leaf gas exchange (LGE) was measured on four fully expanded, mature, and healthy leaf blades from six plants of a treatment between 9.00 am and 11.00 am, using an infrared gas analyzer.

### Soluble sugar (SS) and total soluble protein (TSP) contents in leaves

Soluble sugar content were determined 4 d before termination of experiment following the methodology of Frohlich and Kutschera.^27^ Leaf samples (0.5 g) were put into test tubes containing 10 mL of distilled water, incubated and brought to the level of 25 mL. Of the collected supernatant, 0.5 mL was mixed with 0.5 mL anthrone, 1.5 mL distilled water, and 5 mL sulfuric acid. Solutions were analyzed for SS in a spectrophotometer at 620 nm. Total soluble proteins were evaluated in the leaves following the methodology narrated by Bradford.^28^

### Cadmium concentration in shoot and root

Cadmium concentration in the shoot and root samples was measured using inductively coupled plasma mass spectrometry following Ahmed et al.^29^

### Proline determination

Ninhydrin-oriented methodology was followed to quantify leaf free-proline concentration.^30^ Briefly, 0.5 g fresh sample was put into 10 mL of 3% (w/v) sulfo-salicylic acid. Of this, 2.0 mL solution was added into 2.0 mL of acid ninhydrin (1.26 g ninhydrin +20 mL 6 M ortho-phosphoric acid +30 mL glacial acetic acid) and 2.0 mL of glacial acetic acid. After incubation for 60 min at 80°C, samples were immediately transferred to an ice bath to end the reaction. Toluene (4.0 mL) was then put into the mixture and mixed vigorously. The chromophore was detached from the aqueous phase. Absorbance was measured at 520 nm.

### Oxidative stress markers

The methodology of Dionisio-Sese and Tobita^31^ was followed to evaluate the electrolyte leakage (EL) in leaves. Fresh leaf samples (approximately 200 mg) were chopped separately (<1 cm), placed in distilled water (20 mL) with caps, and incubated in a water bath at 35°C for 2 h. An electrical conductivity meter was used to measure the first electrical conductivity reading (EC1). Each specimen was again autoclaved at 121°C for 20 min and cooled to 25°C for measuring final electrical conductivity (EC2). EL (%) = (EC1/EC2) × 100 was used to calculate the EL. For measuring MDA and H_2_O_2_, fresh samples of 0.5 g of the leaves were taken. MDA and H_2_O_2_ contents were evaluated following the approaches mentioned in Hodges et al.^32^ and Patterson et al.,^33^ respectively.

### Determination of defense enzymes activity

Fresh leaf samples (0.5 g) were homogenized in a chilled mortar and pestle containing 5 mL of ice-cold 50 mM sodium phosphate buffer, pH 7.8, containing 2% (w/v) polyvinylpyrrolidone and 1.0 mM EDTA. The homogenate was then centrifuged at 10,000 g at 4°C for 20 minutes. SOD activity was determined using supernatant stored at 20°C following the methodology by van Rossum et al.^34^ CAT and POD were determined according to Chance and Maehly.^35^ APX activity was determined following Nakano and Asada.^36^ To determine PPO activity, the methodology mentioned in Worthington Enzyme Manual was followed Decker.^37^ The activity of PAL was measured spectrophotometrically following Zucker^38^ and altered by Pendharkar and Nair.^39^ For PAL, 0.3 mL leaf extract was merged with 1.35 mL of 200 μM borate buffer and 1.35 mL of 30 mM of phenylalanine and incubated. In this mixture, 0.2 mL of 5 N HCl was put in order to terminate the reaction. The activity of PAL was noted at 270 nm.

### Statistical analysis

Eight replicates were used in the study. For statistical analysis, one-way analysis of variance (ANOVA) was carried out, followed by LSD test. Graphs were prepared using GraphPad Prism 8.

## Results

### Vegetative traits

**Figure 1.**
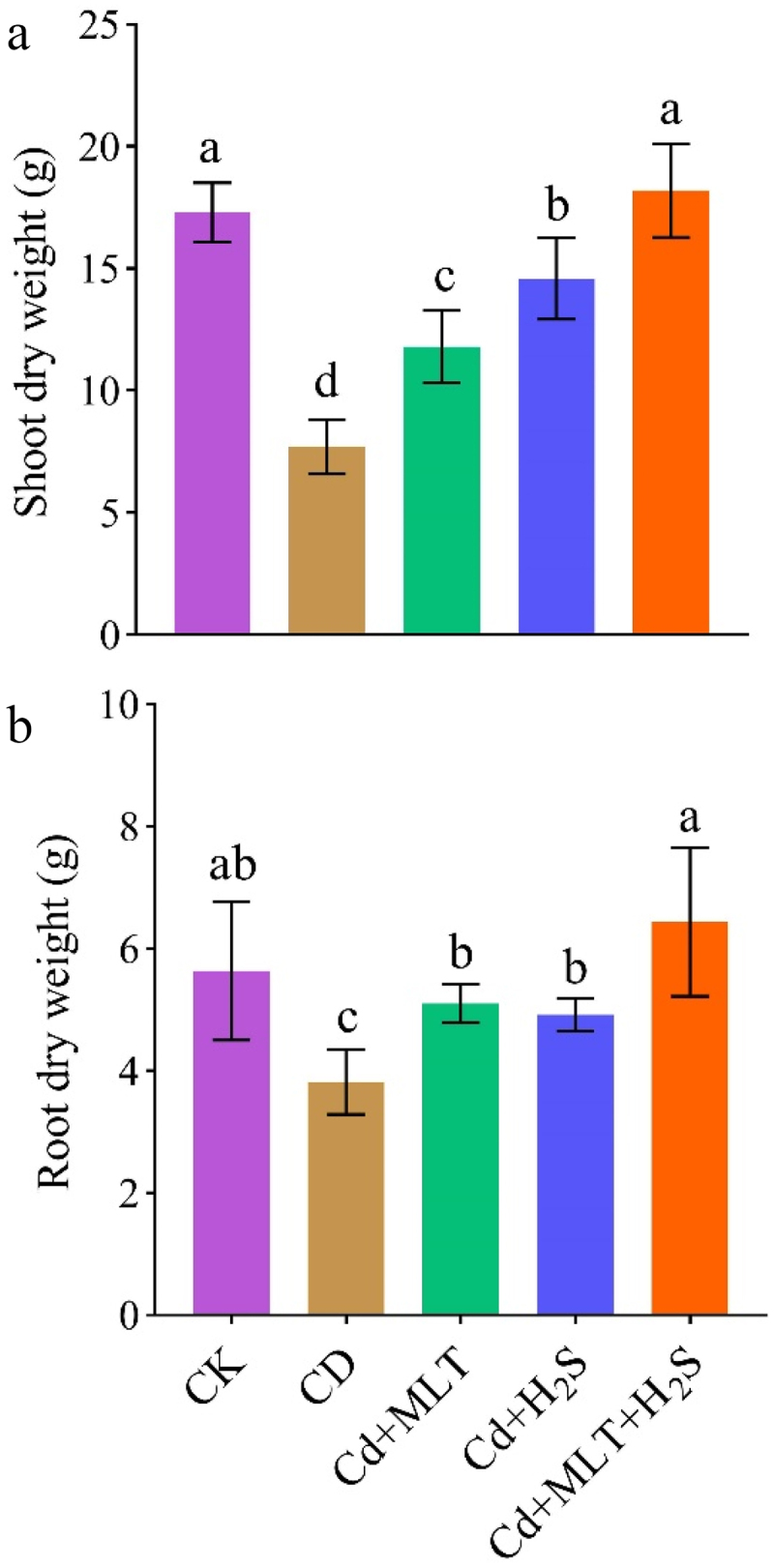
Exogenous MLT (melatonin) and H_2_S (hydrogen sulfide) individual and combined application effect on shoot dry weight (a), and root dry weight (b) of ornamental stock seedlings grown under Cd (cadmium) stress. Data are mean ± SE of six replicates (*n* = 6). Significant differences are revealed via lowercase letters above the bars (at *p* < 0.05), based on LSD test.

Results demonstrated that shoot and root dry weight under Cd stress was significantly lower (55% and 32%) than that of control plants (Figure 1a, b). However, supplementation of MLT, H_2_S alone, or their combination to Cd stressed plants significantly improved the shoot dry weight by 35%, 47% and 58%, respectively, compared with Cd-stressed plants (Figure 1a). Similarly, root dry mass was increased by 25%, 22% and 40% in response to the MLT, H_2_S alone or their combination to stressed plants than control (Figure 1b). Overall, MLT and H_2_S supplementations recovered the Cd-stressed induced reduction in plant growth compared to Cd-stressed control plants.

### Photosynthetic pigments and leaf gas exchange traits

**Figure 2.**
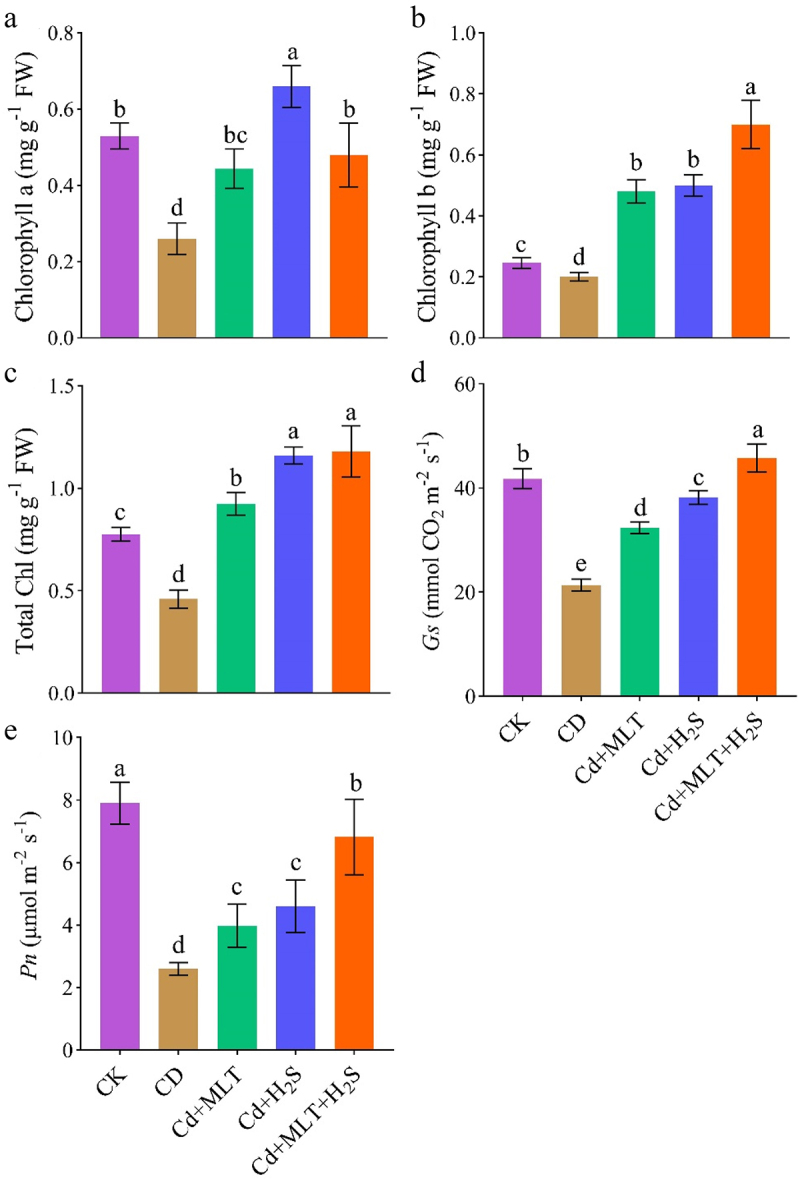
Exogenous MLT (melatonin) and H_2_S (hydrogen sulfide) individual and combined application effect on different photosynthetic pigments and gas exchange parameters (A–E) of ornamental stock seedlings grown under Cd (cadmium) stress. Data are mean ± SE of eight replicates (*n* = 8). Significant differences are revealed via lowercase letters above the bars (at *p* < 0.05), based on LSD test.

Chlorophyll *a*, chlorophyll *b*, and total chlorophyll were markedly decreased by 51%, 19% and 41%, respectively, under Cd stress (Figure 2a–c). Application of MLT, H_2_S and their combination improved the chlorophyll *a* (42%, 61% and 46% respectively), chlorophyll *b* (58%, 60%, and 71% respectively), and total chlorophyll (50%, 61%, and 61% respectively) in comparison to Cd-treated plants (Figure 2a–c).

Leaf gas exchange traits including net photosynthesis rate (Pn) and stomatal conductance (Gs) were markedly decreased by 67% and 49% respectively, under Cd stress (Figure 2d,e). Application of MLT, H_2_S and their combination improved the Pn (35%, 44% and 62% respectively), and Gs (34%, 44%, and 54% respectively), in comparison to Cd-treated stock plants (Figure 2d,e).

### Total soluble sugars, proteins and cadmium uptake

**Figure 3.**
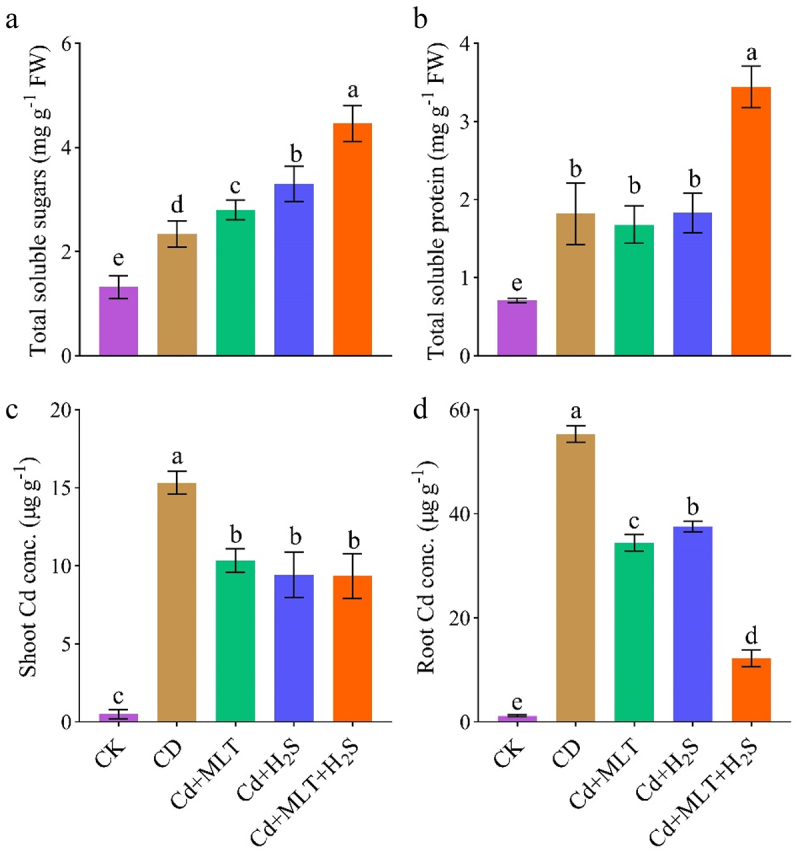
Exogenous MLT (melatonin) and H_2_S(hydrogen sulfide) individual and combined application effect on leaf total soluble sugars (a), total soluble proteins (b), shoot and root cadmium concentration (c, d) of ornamental stock seedlings grown under Cd (cadmium) stress. Data are mean ± SE of eight replicates (*n* = 8). Significant differences are revealed via lowercase letters above the bars (at *p* < 0.05), based on LSD test.

Exposure to Cd significantly increased the level of total soluble sugars and protein compared to non-stressed plants (Figure 3a,b). Consequently, supplementation with MLT, H_2_S further increased the levels of these compared to Cd stressed plants. Combined supplementation with MLT and H_2_S provided the maximum increase (Figure 3a,b). Plants grown under Cd stress had the highest uptake of Cd in both shoot and root tissues (Figure 3c,d). Cd content was significantly decreased in the shoot and roots of plants treated with MLT and H_2_S individual supplementation (Figure 3c,d), and the maximum reduction of Cd uptake in both tissues (52% and 78%) respectively was noted, in response to combined supplementation with MLT and H_2_S (Figure 3c,d). Overall, application of MLT or H_2_S decreased the translocation of Cd in stock plant tissues.

### Oxidative stress markers

**Figure 4.**
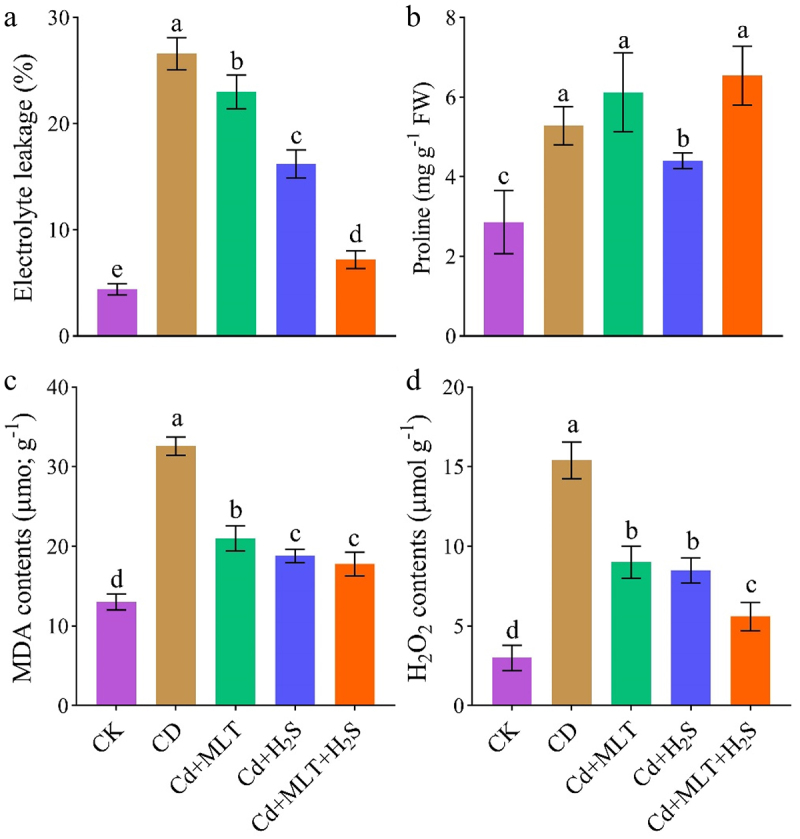
Exogenous MLT (melatonin) and H_2_S (hydrogen sulfide) individual and combined application effect on EL (electrolyte leakage; a), proline b, MDA (malondialdehyde; c), and H_2_O_2_ (hydrogen peroxide; d) of ornamental stock seedlings grown under Cd (cadmium) stress. Data are mean ± SE of eight replicates (*n* = 8). Significant differences are revealed via lowercase letters above the bars (at *p* < 0.05), based on LSD test.

Exposure to Cd significantly increased the level of oxidative stress markers including EL, MDA, and H_2_O_2_ compared to non-stressed plants (Figure 4a,c,d). Supplementation with MLT, H_2_S decreased the level of these oxidative stress markers compared to non-treated plants and combined supplementation with MLT and H_2_S provided the maximum decrease in EL, MDA, and H_2_O_2_ by 73%, 45% and 64%, respectively (Figure 4a,c,d). Proline level was higher in Cd treated plants compared to control (Figure 4b). Supplementation of the combination of MLT and H_2_S further improved the level of proline than the untreated Cd stressed plants (Figure 4b).

### Antioxidant enzyme activities

**Figure 5.**
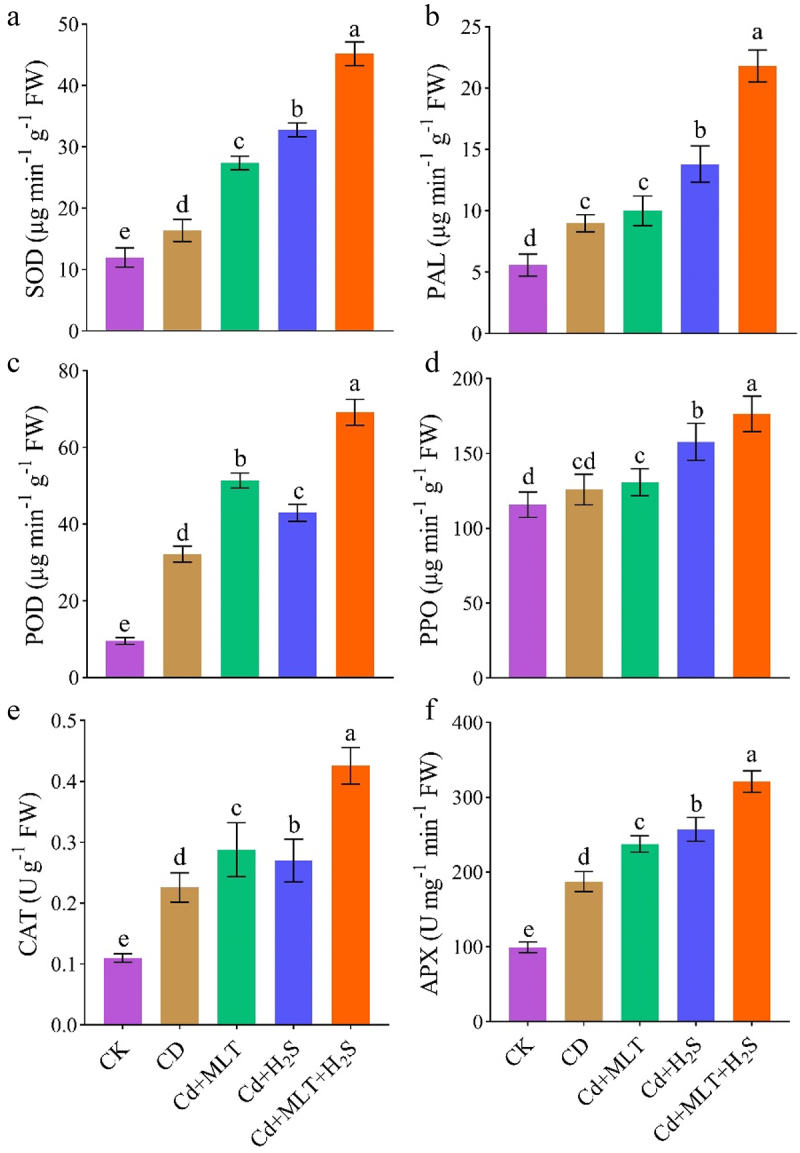
Exogenous MLT (melatonin) and H_2_S (hydrogen sulfide) individual and combined application effect on SOD (superoxide dismutase; a), PAL (phenylalanine ammonia-Lyase; b), POD (peroxidase; c), PPO (polyphenol oxidase; d), CAT (catalase; e), APX (ascorbate peroxidase; f) activity of ornamental stock seedlings grown under Cd (cadmium) stress. Data are mean ± SE of eight replicates (*n* = 8). Significant differences are revealed via lowercase letters above the bars (at *p* < 0.05), based on LSD test.

Defense-related antioxidants including APX, CAT, SOD, PAL, POD, and PPO were determined in stock leaves (Figure 5 a–f). Introduction of Cd to ornamental stock remarkably increased the activity of antioxidants such as SOD by 28%, CAT by 52%, APX by 48%, PAL by 39%, POD by 71%, and PPO by 9%, compared to non-stressed plants (Figure 5a–f). We observed significant increase in the level of SOD (41%, 51% and 65%), PAL (10%, 35% and 59%), POD (37%, 25% and 53%), PPO (4%, 20% and 29%), CAT (23%, 17% and 48%), and APX (22%, 28% and 43%) under MLT, H_2_S and their combined application respectively, as compared with Cd-stressed plants (Figure 5 a–f).

## Discussion

Soils contaminated with trace elements are considered toxic and hazardous for the growth and development of plants.^10^ Cd is extremely noxious, and its presence in agricultural soils has grown due to human activities. Its exposure and uptake in plant cells causes oxidative burst, which disrupts vital cellular functions. The use of phytohormones to increase plant resistance against HMs stress is an effective method that causes no environmental pollution Haider et al.^40^ Melatonin as a powerful biostimulant and antioxidant is considered as a natural treatment to manage HMs stress in plants.^15,16,41,42^ Parallelly, the use of signaling molecules such as H_2_S, is also recognized as a powerful mitigator strategy against HMs stress in plants. During the current investigation, stock plants exposed to Cd stress had lower biomass, which was associated with Cd accumulation, which resulted in chlorophyll degradation. Individual applications of MLT or H_2_S alleviated the Cd-induced decrease in plant biomass and they acted synergistically. The results are similar to the findings of Altaf et al.^43^ and Kaya et al.,^18^ where the authors observed an increase in biomass in response to the individual applications of MLT or H_2_S. Similarly, the application of MLT and H_2_S is reported to enhance the growth of plants grown under Cd stress condition.^44,45^ The improvement in the plant biomass in response to the application of H_2_S and MLT is strongly linked with the antioxidative ability of MLT and the unique properties of H_2_S.

The reduction in the generation of photosynthetic pigments is an early indicator of Cd stress in plants because Cd disrupts the photosynthetic pathway, causing chlorophyll concentration to decrease. Chlorophyll a, chlorophyll b, and total chlorophyll as well as leaf gas exchange traits were reduced significantly in response to Cd stress compared to the control group. Lower pigment concentrations may be due to excessive Cd concentrations in the plants, which disrupted photosynthetic efficiency, resulting in the decrease in photosynthesis in plants (Chen et al. 2023). Supplementation of MLT and H_2_S alleviated the reduction in photosynthetic pigments and leaf gas exchange traits which are vital indicators of photosynthetic efficiency. The use of MLT can assist to preserve the integrity of D1, a significant element of the PSII protein, hence, increasing the photosynthesis (Mukarram et al. 2022; Chen et al. 2023).^43^ In addition, application of these protectants might raise the accumulation of mineral elements or decrease the uptake of Cd that results in improved antioxidants and metabolism resulting in improved photosynthetic efficiency in plants (Chen et al. 2023). This suggests that MLT or H_2_S combined application could be an ameliorative strategy to boost the photosynthetic activity in Cd stressed plants that can increase plant biomass and ultimately crop yield.

Osmoprotectants play a vital role in maintaining water relations and plant metabolism under normal and stressed conditions.^17^ Among the osmoprotectants, proline is considered an important one as it is related to the mitigation of oxidative stress condition in plants.^8^ In the current study, the level of proline increased as a result of Cd stress condition. Supplementation of MLT or H_2_S further boosted the level of proline under Cd exposure. These findings are similar to the studies in which individual application increased the level of proline under Cd stress conditions.^43,46^

Antioxidant defense systems aid plants by scavenging the excessive ROS production in response to abiotic stresses including Cd stress.^8,10^ This system mitigates the Cd induced oxidative burst in plants.^29^ The present study has demonstrated that Cd stress increased the level of oxidative stress markers, revealing oxidative stress in the plants. The application of MLT or H_2_S the level of stress markers and stimulated the activities of antioxidants resulting in enhanced growth. Similar results were observed in previous studies where MLT or H_2_S alone application boosted the activities of antioxidants and reduced the level of oxidative stress markers.^43,46^ These findings indicate that MLT or H_2_S combination can reduce Cd toxicity in stock plant highlighting the potential of these two protectants as a unique alternative for boosting crop output under Cd stress. There still remain considerable gaps in knowledge and application that must be addressed in order to assure the safe use of these protectants for alleviating hazardous HMs in contaminated agricultural soils.

## Conclusion

The current study examined the effect of using MLT or H_2_S together on ornamental stock growth, physiology, and biochemical characteristics under Cd stress. The use of MLT with H_2_S on Cd-stressed plants improved growth metrics, photosynthesis, and overall plant health. The rise in antioxidant enzyme activities and decline in oxidative stress markers suggest that these protectants can alleviate Cd-induced oxidative stress. Furthermore, MLT or H_2_S decreased the Cd accumulation implying their significance in controlling the uptake of Cd. Taken together, these findings show that both MLT or H_2_S have the potential to be a useful strategy in reducing Cd-induced damage in ornamental stock. However, more in-depth research is needed to investigate the detailed mechanisms and to broaden the knowledge related to the possible applications of both MLT or H_2_S for environmental remediation in sustainable ornamental horticulture.

## Contribution statement

Conceptualization, F.Z. and A.M.; formal analysis, F.Z. and A.M.; investigation, F.Z.; data curation, F.Z. and A.M.; writing original draft preparation, F.Z. and A.M.; writing – review and editing, F.Z., A.M., H.M.A., J.H., J.W.H.Y.; project administration, F.Z.; funding acquisition, F.Z. All authors have read and agreed to the published version of the manuscript.

## Data Availability

Data will be made available on the reasonable request to corresponding author.

## References

[1] Nayeri S, Dehghanian Z, Lajayer BA, Thomson A, Astatkie T, Price GW. CRISPR/Cas9-mediated genetically edited ornamental and aromatic plants: a promising technology in phytoremediation of heavy metals. J Clean Prod. 2023;428:139512. doi:10.1016/j.jclepro.2023.139512.

[2] Naing AH, Park DY, Park HC, Kim CK. Removal of heavy metals using iris species: a potential approach for reclamation of heavy metal-polluted sites and environmental beautification. Environ Sci Pollut Res. 2023;30(32):1–9. doi:10.1007/s11356-023-27732-5. 37303013

[3] Lv Y, Zhao Y, He Y, Wang J, Zheng Y, Chen X, Huang F, Liu J, Yu L. Synergistic effects of gamma-aminobutyric acid and melatonin on seed germination and cadmium tolerance in tomato. Plant Signal Behav. 2023;18(1):2216001. doi:10.1080/15592324.2023.2216001. 37302802 PMC10259350

[4] Wang J, Liu W, Wang X, Zeb A, Wang Q, Mo F, Lian Y, Liu J, Yu M, Li J. Assessing stress responses in potherb mustard (brassica juncea var. multiceps) exposed to a synergy of microplastics and cadmium: insights from physiology, oxidative damage, and metabolomics. Sci Total Environ. 2024;907:167920. doi:10.1016/j.scitotenv.2023.167920. 37863229

[5] Khan SR, Ahmad Z, Khan Z, Khan U, Asad M, Shah T. Synergistic effect of silicon and arbuscular mycorrhizal fungi reduces cadmium accumulation by regulating hormonal transduction and lignin accumulation in maize. Chemosphere. 2024;346:140507. doi:10.1016/j.chemosphere.2023.140507. 38303379

[6] Fan P, Wu L, Wang Q, Wang Y, Luo H, Song J, Chen S, Yao H, Chen S. Physiological and molecular mechanisms of medicinal plants in response to cadmium stress: Current status and future perspective. J Hazard Mat. 2023;450:131008. doi:10.1016/j.jhazmat.2023.131008. 36842201

[7] Shiyu QIN, Hongen LIU, Zhaojun NIE, Rengel Z, Wei GAO, Chang LI, Peng ZHAO. Toxicity of cadmium and its competition with mineral nutrients for uptake by plants: a review. Pedosphere. 2020;30(2):168–180. doi:10.1016/S1002-0160(20)60002-9.

[8] Zulfiqar F, Ashraf M. Proline alleviates abiotic stress induced oxidative stress in plants. J Plant Growth Regul. 2022b;42(8):4629–4651. doi:10.1007/s00344-022-10839-3.

[9] Fan P, Wu L, Wang Q, Wang Y, Luo H, Song J, Yang M, Yao H, Chen S. Physiological and molecular mechanisms of medicinal plants in response to cadmium stress: Current status and future perspective. J Hazard Mater. 2023;450:131008. doi:10.1016/j.jhazmat.2023.131008. 36842201

[10] Zulfiqar F, Ashraf M. Antioxidants as modulators of arsenic-induced oxidative stress tolerance in plants: an overview. J Hazard Mat. 2022a;427:127891. doi:10.1016/j.jhazmat.2021.127891. 34848065

[11] Khan MN, Siddiqui MH, Mukherjee S, AlSolami MA, Alhussaen KM, AlZuaibr FM, Alsubaie QD, Al-Amri AA, Alsubaie QD. Melatonin involves hydrogen sulfide in the regulation of H±ATPase activity, nitrogen metabolism, and ascorbate-glutathione system under chromium toxicity. Environ Pollut. 2023;323:121173. doi:10.1016/j.envpol.2023.121173. 36740162

[12] Zhao Y, Wang Q, Gu D, Huang F, Liu J, Yu L, Yu X. Melatonin, a phytohormone for enhancing the accumulation of high-value metabolites and stress tolerance in microalgae: applications, mechanisms, and challenges. Bioresour Technol. 2023;130093:130093. doi:10.1016/j.biortech.2023.130093. 38000641

[13] Song C, Manzoor MA, Mao D, Ren X, Zhang W, Zhang Y. Photosynthetic machinery and antioxidant enzymes system regulation confers cadmium stress tolerance to tomato seedlings pretreated with melatonin. Sci Hortic (Amsterdam). 2024;323:112550.

[14] Arnao MB, Hernández‐Ruiz J. Melatonin as a regulatory hub of plant hormone levels and action in stress situations. Plant Biol. 2021;23:7–19. 33098247 10.1111/plb.13202

[15] Song C, Manzoor MA, Mao D, Ren X, Zhang W, Zhang Y. Photosynthetic machinery and antioxidant enzymes system regulation confers cadmium stress tolerance to tomato seedlings pretreated with melatonin. Sci Hortic (Amsterdam). 2024;323:112550. doi:10.1016/j.scienta.2023.112550.

[16] Yang H, Fang R, Luo L, Yang W, Huang Q, Yang C, Wang J, Gong W, Wang J. Potential roles of melatonin in mitigating the heavy metals toxicity in horticultural plants. Sci Hortic (Amsterdam). 2023;321:112269. doi:10.1016/j.scienta.2023.112269.

[17] Zulfiqar F, Akram NA, Ashraf M. Osmoprotection in plants under abiotic stresses: new insights into a classical phenomenon. Planta. 2020;251(1):1–17. doi:10.1007/s00425-019-03293-1. 31776765

[18] Kaya C, Ugurlar F, Ashraf M, Alyemeni MN, Bajguz A, Ahmad P. The involvement of hydrogen sulphide in melatonin-induced tolerance to arsenic toxicity in pepper (capsicum annuum L.) plants by regulating sequestration and subcellular distribution of arsenic, and antioxidant defense system. Chemosphere. 2022;309:136678. doi:10.1016/j.chemosphere.2022.136678. 36191761

[19] Khan MN. S-nitrosoglutathione-facilitated activation of ATP synthase involves hydrogen sulfide during the response of plants to cadmium toxicity. S Afr J Bot. 2024;165:176–187. doi:10.1016/j.sajb.2023.12.033.

[20] Haghi V, Namdjoyan S, Soorki AA. Interactive effects of exogenous melatonin and hydrogen sulfide in alleviating lead toxicity in safflower seedlings. Ind Crops Prod. 2022;187:115523. doi:10.1016/j.indcrop.2022.115523.

[21] McLean E. Soil pH and lime requirement. In: Page, A.L., Miller, R.H., Keeney, D.R., editor(s). Methods of Soil Analysis. Part 2, 2nd ed. Agronomy Monograph, 9. Madison: American Society of Agronomy and Soil Science Society of America. 1982;199–224.

[22] Page AL, Miller RH, Keeny DR. Methods of soil analysis (part 2). Chem. Microbiol. Proper. Agron 9. 1982;9.

[23] Bouyoucos GJ. Hydrometer method improved for making particle size analyses of soils 1. Agron J. 1962;54(5):464–465. doi:10.2134/agronj1962.00021962005400050028x.

[24] Walkley A, Black IA. An examination of the Degtjareff method for determining soil organic matter, and a proposed modification of the chromic acid titration method. Soil Sci. 1934;37(1):29–38. doi:10.1097/00010694-193401000-00003.

[25] Soltanpour PN. Use of ammonium bicarbonate DTPA soil test to evaluate elemental availability and toxicity. Commun Soil Sci Plant Anal. 1985;16(3):323–338. doi:10.1080/00103628509367607.

[26] Lichtenthaler HK. Chlorophylls and carotenoids: pigments of photosynthetic biomembranes. In Methods in enzymology. Academic Press. 1987;148:350–382.

[27] Frohlich M, Kutscherah U. Changes in soluble sugars and proteins during. Development of rye coleoptiles. J Plant Physiol. 1995;146(1–2):121–125. doi:10.1016/S0176-1617(11)81977-2.

[28] Bradford MM. A rapid and sensitive method for the quantitation of microgram quantities of protein utilizing the principle of protein-dye binding. Analytical Biochem. 1976;72(1–2):248–254. doi:10.1016/0003-2697(76)90527-3. 942051

[29] Ahmed T, Masood HA, Noman M, AA A-H, Alghanem SM, Khan MM, Li B, Manzoor N, Rizwan M, Qi X. Biogenic silicon nanoparticles mitigate cadmium (Cd) toxicity in rapeseed (*Brassica napus* L.) by modulating the cellular oxidative stress metabolism and reducing Cd translocation. J Hazard Mat. 2023;132070. doi:10.1016/j.jhazmat.2023.132070. 37478591

[30] Bates LS, Waldren RP, Teare ID. Rapid determination of free proline for water-stress studies. Plant Soil. 1973;39(1):205–207. doi:10.1007/BF00018060.

[31] Dionisio-Sese ML, Tobita S. Antioxidant responses of rice seedlings to salinity stress. Plant Sci. 1998;135(1):1–9. doi:10.1016/S0168-9452(98)00025-9.

[32] Hodges DM, JM D, Forney CF, Prange RK. Improving the thiobarbituric acid-reactive-substances assay for estimating lipid peroxidation in plant tissues containing anthocyanin and other interfering compounds. Planta. 1999;207(4):604–611. doi:10.1007/s004250050524. 28456836

[33] Patterson BD, EA M, Ferguson IB. Estimation of hydrogen peroxide in plant extracts using titanium (IV). Analyt Biochem. 1984;139(2):487–492. doi:10.1016/0003-2697(84)90039-3. 6476384

[34] van Rossum MW, Alberda M, van der Plas LH, van Rossum MWPC, van der Plas LHW. Role of oxidative damage in tulip bulb scale micropropagation. Plant Sci. 1997;130(2):207–216. doi:10.1016/S0168-9452(97)00215-X.

[35] Chance B, Maehly AC. Assay of catalases and peroxidases. Methods Enzymol. 1955;2:764–817. 10.1002/9780470110171.ch14 13193536

[36] Nakano Y, Asada K. Hydrogen peroxide is scavenged by ascorbate-specific peroxidase in spinach chloroplasts. Plant Cell Physiol. 1981;22:867–880.

[37] Decker A. Enzymes reagents and related enzyme biochemicals. Worthingto n enzyme manual. Worthington biochemical corporations freehold. New Jersey, USA; 1977. pp. 74–76.

[38] Zucker M. Sequential induction of phenylalanine ammonia-lyase in cell suspension culture of barley. Plant Physiol. 1965;5:822–827. 10.1104/pp.55.5.822 PMC541717 16659175

[39] Pendharkar MB, Nair PM. Induction of phenylalanine ammonia lyase (PAL) in gamma irradiated potatoes. Radiation Bot. 1975;15(2):191–197. doi:10.1016/S0033-7560(75)80007-X.

[40] Haider FU, Farooq I, Khan M, Cai L, Li Y, Cai L, Li Y. Co-application of biochar and plant growth regulators improves maize growth and decreases Cd accumulation in cadmium-contaminated soil. J Cleaner Prod. 2024;140515. doi:10.1016/j.jclepro.2023.140515.

[41] Awan SA, Khan I, Rizwan M, Irshad MA, Xiaosan W, Zhang X, Huang L. Reduction in the cadmium (cd) accumulation and toxicity in pearl millet (*Pennisetum glaucum* L.) by regulating physio-biochemical and antioxidant defense system via soil and foliar application of melatonin. Environ Pollut. 2023;328:121658. doi:10.1016/j.envpol.2023.121658. 37075919

[42] Huang J, Jing HK, Zhang Y, Chen SY, Wang HY, Cao Y, Zhu XF, Lu YH, Zheng QS, Shen RF. Melatonin reduces cadmium accumulation via mediating the nitric oxide accumulation and increasing the cell wall fixation capacity of cadmium in rice. J Hazard Mat. 2023;445:130529. doi:10.1016/j.jhazmat.2022.130529. 37055957

[43] Altaf MA, Hao Y, Shu H, Mumtaz MA, Cheng S, Alyemeni MN, Wang Z, Wang Z. Melatonin enhanced the heavy metal-stress tolerance of stock by mitigating the oxidative damage and reducing the heavy metal accumulation. J Hazard Mat. 2023;454:131468. doi:10.1016/j.jhazmat.2023.131468. 37146338

[44] Saqib M, Shahzad U, Zulfiqar F, Tiwari RK, Lal MK, Naz S, Altaf MA, Awan ZA, El-Sheikh MA, Altaf MA. Exogenous melatonin alleviates cadmium-induced inhibition of growth and photosynthesis through upregulating antioxidant defense system in strawberry. South African J Bot. 2023;157:10–18. doi:10.1016/j.sajb.2023.03.039.

[45] Zheng X, Zhang B, Pan N, Cheng X, Lu W. Hydrogen sulfide alleviates cadmium stress by enhancing photosynthetic efficiency and regulating sugar metabolism in wheat seedlings. Plants. 2023;12(13):2413. doi:10.3390/plants12132413. 37446974 PMC10346985

[46] Yusuf M, Saeed T, Almarri HJ, Khan TA, Faizan M, Elsayed N. Hydrogen sulfide counteract copper induced inhibition of photosynthetic performance through altered proline metabolism and enhanced antioxidants in *Cucumis sativus*. Plant Stress. 2023;10:100222. doi:10.1016/j.stress.2023.100222.

